# Supplementary material for: WordCommentsAnalyzer: A windows software tool for qualitative research
Source: F1000Res. 2018 Sep 4;7:536. Originally published 2018 May 3. [Version 2] doi: 10.12688/f1000research.14819.2 (PMC6137411; doi:10.12688/f1000research.14819.2)
Supplement: Supplementary file 3 [file f1000research-7-17614-s0002.tgz › b2a79496-4ada-47c4-9682-a99ce867836d.zip!/Eight Iranian Health Journals/Salmand_Iranian Journal of Ageing _28.docx]

© Salmand_Iranian Journal of Ageing . This work is licensed under CC BY-NC 4.0 (https://creativecommons.org/licenses/by-nc/4.0/).

Volume & Issue: دوره 6 - شماره 2

Title: پیش‌بینی ابتلا به کمبود ویتامین D در سالمندان و ساکنان خانه‌های سالمندان شهر تبریز با استفاده از مدل رگرسیون استریوتایپ

http://salmandj.uswr.ac.ir/article-1-418-fa.html

اهداف: کمبود ویتامین D یک مشکل مهم بهداشتی جامعه است که در افراد مسن شایع‌تر است و ممکن است در افراد مسن ساکن خانه سالمندان نیز بیشتر باشد. تا کنون مطالعات متعددی بر روی کمبود ویتامین D با استفاده از مدل‌های آماری موجود انجام شده است. در این مطالعه، به منظور یافتن عوامل خطر ساز مرتبط با کمبود ویتامین D در افراد مسن ساکن خانه سالمندان و مقایسه آن با افراد مسن خارج خانه سالمندان از روش رگرسیون بخت‌های متناسب و رگرسیون استریوتایپ استفاده شد. مواد و روش‌ها: در یک مطالعه مورد-شاهدی با 140 نفر سالمند ساکن خانه سالمندان و 140 نفر سالمند خارج از خانه سالمندان وارد مطالعه شدند. در این مطالعه متغیر سطوح سرمی 25(OH)D به عنوان متغیر پاسخ، و متغیرهای سن، جنس، نمایه توده بدن، میزان مواجهه با نور آفتاب به عنوان متغیرهای پیشگو برای کمبود ویتامین D بررسی شدند. تحلیل‌های مربوط با استفاده از روش‌های رگرسیون بخت-های متناسب و رگرسیون استریوتایپ و برآورد پارامترهای این دو مدل انجام شد. برای ارزیابی و مقایسه دو مدل مذکور از آماره‌ی انحراف (AIC) استفاده شد. نرم افزار Stata.9/1 برای انجام تحلیل‌ها مورد استفاده قرار گرفت. یافته‌ها: میانگین سطح سرمی 25(OH)D در افراد ساکن خانه سالمندان ng/ml 16/10±16/65 و در افراد خارج از خانه سالمندان ng/ml 39/62±24/78 بود (P=0/001). شیوع کمبود ویتامین D (کمتر از ng/ml 20) در گروه ساکن خانه سالمندان %75 و در گروه خارج خانه سالمندان %23/87 افراد را شامل شد. با استفاده از مدل‌های رگرسیون بخت‌های متناسب و رگرسیون استریوتایپ، متغیرهای جنس، سن، نمایه توده بدن، میزان مواجهه با نور آفتاب و عضویت خانه سالمندان، برازش داده شد در هر دو مدل مربوطه متغیرهای گروه و میزان مواجهه با نورآفتاب معنادار شدند (P<0/001). مدل رگرسیون استریوتایپ شامل متغیر گروه (نسبت بخت برای رسته کمبود شدید برابر با 42/85 و (CI95%:9.93-185.67) و متغیر میزان مواجهه با نور آفتاب نور آفتاب (نسبت بخت برای رسته کمبود شدید برابر با 0/717 و (CI95%: 0/642-0/792) بود. نتیجه‌گیری: در این مطالعه شیوع بالایی از کمبود ویتامین D در افراد سالمند خانه سالمندان نشان داده شد که میزان آن در مقایسه با افراد خارج از خانه سالمند بیشتر بود. به نظر می‌رسد رگرسیون استریوتایپ به عنوان یک روش جدید، در بین مدل‌های لوجستیک چندرسته‌ای، قابلیت خوبی برای غربالگری انواع بیماری از جمله بیماری کمبود ویتامین D دارد.

Title: تأثیر تمرینات ویبریشن تمام بدن و دوره‌های بی‌تمرینی بر عملکرد عصبی عضلانی مردان سالمند

http://salmandj.uswr.ac.ir/article-1-420-fa.html

اهداف: هدف از انجام مطالعه حاضر بررسی تأثیر هشت هفته تمر ینات ویبریشن تمام بدن و دوره بی تمرینی بر عملکرد عصبی عضلانی مردان سالمند سالم بود. مواد و روش‌ها: 30 مرد سالمند (9/6±70) دردو گروه 15 نفره تمرینات ویبریشن تمام بدن و گروه کنترل قرار گرفتند. قبل و پس از انجام هشت هفته تمرینات و بعد از چهار، شش و هشت هفته بی تمرینی آزمون‌های 5- به عمل آمد که آزمون‌هایی برای ارزیابی عملکرد عصبی -عضلانی در افراد Time Up & Go و  Chair Stand-5  سالمند می‌باشند. یافته‌ها: نتایج روش های آماری تحلیل واریانس با اندازه گیری مکرر و تحلیل واریانس یک راهه نشان دادند که پس از انجام تمرینات، عملکرد عصبی-عضلانی آزمودنی ها در گروه تمرینات ویبریشن تمام بدن بطور معنی داری بهبود یافت (P≤0/05). همچنین در گروه تمرین ویبریشن تمام بدن بین پس‌آزمون و شش و هشت هفته بی‌تمرینی اختلاف معنی‌داری مشاهده شد (P≤0/05). نتیجه‌گیری: با توجه به نتایج بدست آمده می‌توان گفت که تمرینات ویبریشن تمام بدن می‌توانند تأثیر معنی‌داری بر عملکرد عصبی عضلانی افراد سالمند داشته باشند و احتمال افتادن و به زمین خوردن سالمندان را کاهش دهند. ضمن اینکه اثرات این تمرینات ماندگار نیستند و پس از شش هفته بی تمرینی به سطح قبل از تمرینات رسیده‌اند و این احتمال وجود دارد که بتوان تمرینات ویبریشن تمام بدن را به عنوان یک تمرین تعادلی ایمن برای سالمندان توصیه کرد.

Title: کیفیت زندگی سالمندان روستایی: مطالعه‌ای در شهرستان دشتی استان بوشهر

http://salmandj.uswr.ac.ir/article-1-419-fa.html

اهداف: امید به زندگی در دنیا افزایش یافته و به‌طور روز افزونی بر شمار سالمندان افزوده می‌شود. اکنون تنها زنده بودن هدف نیست بلکه چگونگی زندگی نیز مورد توجه است. سالمندان روستایی نیز از این امر مستثنی نیستند. باید کیفیت زندگی آن‌ها بررسی شده تا تصمیم‌گیری‌های مناسب براساس نتایج به دست آمده، اتخاذ گردد. هدف این پژوهش بررسی کیفیت زندگی سالمندان روستایی است. مواد و روش‌ها: پژوهش حاضر یک مطالعه مقطعی توصیفی تحلیلی بوده که کیفیت زندگی سالمندان روستایی مورد بررسی SF شهرستان دشتی استان بوشهر را در بهمن‌ماه سال 1388 با استفاده از پرسشنامه کیفیت زندگی 36 قرار داده است. با استفاده از روش نمونه‌گیری خوشه‌ای چند مرحله‌ای، 69 سالمند روستایی که ملاک‌های ورود جهت تحلیل داده‌ها SPSS به مطالعه را دارا بودند، به عنوان نمونه پژوهش برگزیده شدند. از نرم افزار آماری 17 مستقل و تحلیل واریانس یکطرفه جهت بررسی رابطه متغیرهای دموگرافیک با t استفاده شد. همچنین از آزمون کیفیت زندگی استفاده گردید. یافته ها: در بررسی رابطه کیفیت زندگی با متغیرهای دموگرافیک مشخص گردید که در دو قلمرو سرزندگی وسلامت روان باتوجه به سن و وضعیت تأهل، بین سالمندان تفاوت وجود دارد. (P<0/05) تفاوت آماری معناداری در کیفیت زندگی سالمندان مرد و زن مشاهده نشد (P>0/05) میانگین نمرات سالمندان با سواد قرآنی در قلمرو نقش فیزیکی، بالاتر از سالمندان بی‌سواد بود (0/016=P). همچنین مانگین نمرات کیفیت زندگی، ابعاد و قلمروهای آن به جز قلمرو عملکرد اجتماعی، پایین تر از متوسط بود. نتیجه‌گیری: کیفیت زندگی سالمندان روستایی شهرستان دشتی پایین تر از متوسط است به‌طوری که احتیاج به رسیدگی فوری داشته و انجام مداخلاتی جهت بهبود کیفیت زندگی آن‌ها ضروری به نظر می‌رسد.

Title: سالمندی و مشکلات سلامتی: یک مطالعه مقطعی در شهرستان شاهرود

http://salmandj.uswr.ac.ir/article-1-417-fa.html

اهداف: سالمندی در تمامی جوامع به عنوان یک مسئله مهم مطرح می‌باشد. در کشور ما نیز با توجه به روند و هرم جمعیتی برنامه-ریزی جهت هرگونه خدمات، نیازمند شناخت نیازهای واقعی سالمندان است. مطالعه با هدف تعیین فراوانی مشکلات سلامتی سالمندان شهرستان شاهرود در سال 1388 انجام شد. مواد و روش‌ها: این پژوهش از نوع مطالعات کاربردی است که به شیوه مقطعی برروی 1046 نفر از سالمندان 69-60 ساله شهری و روستایی شهرستان شاهرود انجام شد. ابزار جمع‌آوری داده‌ها پرسشنامه 33 سئوالی بوده که از طریق مصاحبه، معاینه و توزین سالمندان تکمیل گردید. تجزیه و تحلیل داده‌ها با استفاده از آزمون‌های تحلیلی آماری و به کمک نرم افزار SPSS انجام شد. یافته‌ها: %61 سالمندان مورد مطالعه زن بودند. %59/8 در مناطق شهری سکونت داشتند. %60 بیسواد و %13 از آنان تنها زندگی می‌کردند. %44/7 از سالمندان مشکلات خواب، %23 مشکلات شنوایی، %62  مشکلات بینایی، %45/2 مشکلات پا، %21/7 یبوست، %45/3 اختلال حافظه، %14/8 مشکلات دستگاه ادراری، %90/8 مشکلات دهان ودندان و %43 استرس را گزارش کردند. بین جنس با دردهای مفصلی و اختلالات خواب، محل زندگی با اختلالات بینایی، تحصیلات با اختلالات بینایی و اختلالات خواب و مشکلات دهان و دندان ارتباط معنی‌دار آماری مشاهده شد (0/05>P). نتیجه‌گیری: مشکلات دهان و دندان، بینایی، پا و حافظه از مهمترین مشکلات سالمندان بوده که توسعه مراقبت‌های نظام‌یافته و ارایه خدمات مناسب به سالمندان در قالب شبکه‌های بهداشتی‌درمانی و مراقبت‌های بهداشتی اولیه، افزایش آگاهی‌های بهداشتی سالمندان و آموزش مهارت‌های زندگی به آنان و خانواده‌هایشان می تواند نقش موثری در بهبود وضعیت سالمندان داشته باشد.

Title: اثر ورزش و فعالیت بدنی بر میزان شادی زنان یائسه

http://salmandj.uswr.ac.ir/article-1-415-fa.html

اهداف: هدف پژوهش حاضر بررسی اثر ورزش و فعالیت بدنی بر میزان شادی زنان یائسه است. مواد و روش‌ها: پژوهش حاضر از نوع تجربی است.جامعه آماری تحقیق زنان سالمند شهر شیراز بودند که از طریق فراخوان محلی به شرکت در پژوهش دعوت شده بودند. از بین داوطلبان شرکت در تحقیق 72 نفر با توجه به ملاک های ورود (عدم ابتلا به بیماری، دامنه سنی مناسب، عدم مصرف منظم دارو و علاقمندی و داشتن وقت کافی در زمان های موردنظر شرکت در تحقیق) انتخاب شده و با انتساب تصادفی در سه گروه تمرینی (پیاده‌روی، حرکت در آب و تمرین با وزنه) و یک گروه کنترل (هر گروه 18 نفر) قرار گرفتند. سه گروه تمرینی به مدت دو ماه در روزها و ساعت‌های مشابه تحت نظر مربی با شدت و مدت معین تمرینات خود را انجام ‌دادند. زمان و نوع اجزای عمومی فعالیت (گرم کردن و سرد کردن) برای سه گروه مشابه و یکسان بود. مدت زمان جزء اختصاصی تمرین از نظر زمان برای هر سه گروه یکسان ولی از لحاظ محتوا به اشکال گوناگون (پیاده‌روی، حرکت در آب و تمرین با وزنه) انجام شد. برنامه تمرینی از نظر زمان و شدت تمرین به صورت فزاینده اجرا گردید، به طوری که در روز اول مدت زمان کل تمرین 50 دقیقه و روز آخر 80 دقیقه به طول انجامید. برای سنجش شادی از پرسشنامه تجدید نظر شده شادی آکسفورد استفاده شد که قبل و بعد از 8 هفته برنامه تمرینی، توسط آزمودنی ها تکمیل گردید. نتایج با آنالیز واریانس یک طرفه و نرم افزار SPSS تجزیه و تحلیل شد. یافته‌ها: نتایج بیانگر تفاوت معنی‌دار بین گروه‌های ورزشی با گروه کنترل بود. یافته‌های تحقیق نشان دادند که شرکت در فعالیت‌های ورزشی تاثیر معنی‌داری بر میزان شادی زنان دارد و گروه تمرین با وزنه، از میان گروه‌های تمرینی، بیشترین تاثیر را نسبت به گروه کنترل نشان داد. نتیجه‌گیری: شرکت در برنامه‌های ورزشی باعث پیشرفت در کلیه ابعاد شادی در گروه‌های ورزشی تحقیق شد. طرح‌ریزی برنامه تمرینی منظم می‌تواند برای زنان مسن مفید باشد و این گروه آسیب‌پذیر می‌توانند از برنامه‌های تمرینی جهت کاهش عوارض دوران یائسگی استفاده کنند.

Title: عفونت‌های شایع در افراد سالمند بستری در بخش عفونی بیمارستان شهید بهشتی کاشان

http://salmandj.uswr.ac.ir/article-1-422-fa.html

اهداف: استعداد ابتلا به عفونت‌ها در سالمندان افزایش می‌یابد. با توجه به بالا بودن میزان عفونت‌ها، و مرگ‌ومیر و ازکارافتادگی حاصل از آن‌ها و علائم متفاوت عفونت‌ها در سالمندان که می‌تواند منجربه تأخیر در تشخیص گردد، این مطالعه به منظور بررسی علائم بالینی و یافته‌های آزمایشگاهی بیماری‌های عفونی در سالمندان بستری در بخش عفونی بیمارستان شهید بهشتی کاشان طراحی گردید. مواد و روش‌ها: این مطالعه توصیفی بر روی 248 نفر از سالمندان مبتلا به بیماری عفونی بستری شده در بیمارستان شهید بهشتی کاشان در سال 88 انجام گرفت. ملاک تشخیص اولیه بیماری عفونی، یافتن تب یا کانون مشخصی از عفونت در معاینه بود. از تمام بیماران شرح حال و معاینه فیزیکی به عمل آمد و بررسی‌های آزمایشگاهی از جمله و همچنین تصویربرداری تشخیصی از جمله سونوگرافی، سی تی اسکن CRP و ESR ،CBC، کشت خون و ادرار انجام شد. سپس پرسشنامه‌ای حاوی اطلاعات دموگرافیک، علائم بالینی و یافته‌های آزمایشگاهی از طریق MRI و مصاحبه و مرور پرونده بیماران تکمیل و نتایج به صورت آمار توصیفی ارائه گردید. یافته‌ها: در این مطالعه از مجموع 248 بیمار شرکت‌کننده، 133 نفر (%53/6) سال قرار داشتند. میانگین سنی بیماران 74/3 سال بود که بیشتر آن‌ها %48/4 درگروه سنی 75-60 سال قرار داشتند. شایع ترین شکایات اصلی بیماران تب (%19/4) و تنگی نفس %14/9 و شایع‌ترین بیماری زمینه ای فشار خون بالا بود (%37). شایع‌ترین بیماری عفونی پنومونی (%23/4) و سپس سپیس بود (%15/7) بود. نتیجه‌گیری: شایع‌ترین بیماری عفونی منجر به بستری و مرگ در سالمندان پنومونی و سپسیس می‌باشد.

Title: رابطه ترکیبی مؤلفه‌های نشانگان بازنشستگی با نشانه‌های سلامت عمومی در سالمندان بازنشسته

http://salmandj.uswr.ac.ir/article-1-416-fa.html

اهداف: هدف از این مطالعه، بررسی رابطه مولفه‌های نشانگان بازنشستگی با نشانه‌های سلامت عمومی در سالمندان بازنشسته شهر اصفهان بود. مواد و روش‌ها: این مطالعه به روش توصیفی و همبستگی انجام شد. جامعه آماری پژوهش را سالمندان بازنشسته شهر اصفهان تشکیل دادند، که از میان آن‌ها به شیوه‌ تصادفی طبقه‌ای، 461 نفر برای شرکت در پژوهش انتخاب و پرسشنامه‌های نشانگان بازنشستگی (درماندگی و شکست، پیری و بطالت، تلاش و جهت‌گیری جدید و سردرگمی و تعارض) و سلامت عمومی (نشانه‌ها و شکایات جسمانی، اضطراب و بیخوابی، نارسایی عملکرد اجتماعی و افسردگی) در مورد آن‌ها اجرا شد. یافته‌ها: 1. برای پیش‌بینی نشانه‌ها و شکایات جسمانی، سه مؤلفه احساس درماندگی و شکست، احساس پیری و بطالت و احساس تلاش و جهت‌گیری جدید دارای توان پیش‌بین معنادار بودند؛ 2. برای اضطراب و بیخوابی، احساس درماندگی و شکست و احساس تلاش و جهت‌گیری و احساس پیری و بطالت دارای توان پیش‌معنادار بودند؛ 3. برای نارسایی عملکرد اجتماعی، احساس درماندگی و شکست و احساس تلاش و جهت‌گیری جدید دارای توان پیش‌معنادار بودند؛ 4. برای افسردگی نیز احساس درماندگی و شکست و احساس تلاش و جهت‌گیری جدید دارای توان پیش‌بینی معنادار بودند. نتیجه‌گیری: نتایج این پژوهش نشان داد که احساس درماندگی و شکست و احساس تلاش و جهت‌گیری جدید، دو مؤلفه‌های اصلی هستند که در سالمندانه بازنشسته باید بطور جدی مورد توجه قرار گیرد. بنظر می‌رسد که در این دو حوزه لازم است مراکز مشاوره وابسته به کانون‌های بازنشستگی تأسیس و سالمندانه بازنشسته را یاری کنند.

Title: مقایسه تأثیر تمرین در آب و بیرون از آب بر تعادل ایستا و پویای مردان سالمند سالم

http://salmandj.uswr.ac.ir/article-1-421-fa.html

اهداف: مطالعه حاضر به منظور مقایسه تأثیر تمرین در آب و بیرون از آب بر کنترل تعادل م ردان سالمند سالم انجام شد. مواد و روش‌ها: این مطالعه یک تحقیق نیمه تجربی با طرح پی شآزمون پس آزمون و با دو گروه مداخله ی تمرینی و یک گروه کنترل می باشد، که در آن 40 مرد سالمند سالم بالای 65 سال به صورت تصادفی به سه گروه تمرین در آب (13 نفر)، تمرین در بیرون از آب (12 نفر) و کنترل (15 نفر) تقسیم شدند. دو گروه تمرین در آب و بیرون از آب هر کدام به مدت 6 هفته، هر هفته سه جلسه و هر جلسه به مدت 60 دقیقه، در تمرینات مشابه در دو محیط آب و بیرون از آب شرکت کردند. به گروه کنترل هیچ‌گونه تمرین خاصی داده نشد و به آن‌ها توصیه شد فقط فعالیت‌های معمولی روزانه خود را انجام دهند. تغییرات در تعادل ایستا و پویای آزمودنی ها، قبل و بعد از دوره تمرین، به‌وسیله آزمون‌های ستاره و شارپند رومبرگ اندازه‌گیری شد. تجزیه و تحلیل آماری، با استفاده از آزمون‌های آماری مناسب در سطح معنی‌داری 0/05>P با استفاده از نرم‌افزار SPSS (نسخه 16) انجام شد. یافته‌ها: نتایج تحقیق نشان داد که اعمال برنامه تمرین در محیط آب و بیرون از آب می‌تواند باعث بهبود تعادل ایستا (P=0/005) و پویا (P=0/001) در مردان سالمند شود. همچنین تفاوت معنی‌داری بین داده‌های پس‌آزمون آزمودنی‌های گروه تمرین در آب و بیرون از آب با گروه کنترل در آزمون‌های تعادل ایستا و پویا دیده شد (P=0/05). با این حال تفاوت معناداری بین نمرات پیش‌آزمون و پس‌آزمون گروه کنترل مشاهده نشد (P=0/05). همچنین تفاوت معنی‌داری بین دو گروه تمرین در آب و بیرون از آب در آزمون‌های تعادل ایستا (P=0/5) و پویا (P=0/6) دیده نشد. نتیجه‌گیری: براساس یافته های این پژوهش، به کارگیری تمرینات در آب و بیرون از آب منجر به بهبود تعادل ایستا و پویا در سالمندان می‌شود؛ با این حال تفاوتی در استفاده از هر کدام از آن‌ها وجود ندارد .مطالعات بیشتری با حجم نمونه بالاتر و در گروه‌های مختلف سالمندان جهت تعیین تأثیر طولانی مدت این تمرینات پیشنهاد می‌شود.
